# Enantioseparation of 5,5′-Dibromo-2,2′-dichloro-3-selanyl-4,4′-bipyridines on Polysaccharide-Based Chiral Stationary Phases: Exploring Chalcogen Bonds in Liquid-Phase Chromatography

**DOI:** 10.3390/molecules26010221

**Published:** 2021-01-04

**Authors:** Paola Peluso, Alessandro Dessì, Roberto Dallocchio, Barbara Sechi, Carlo Gatti, Bezhan Chankvetadze, Victor Mamane, Robin Weiss, Patrick Pale, Emmanuel Aubert, Sergio Cossu

**Affiliations:** 1 Institute of Biomolecular Chemistry ICB, CNR, Secondary Branch of Sassari, Traversa La Crucca 3, Regione Baldinca, Li Punti, 07100 Sassari, Italy; alessandro.dessi@cnr.it (A.D.); roberto.dallocchio@cnr.it (R.D.); barbara.sechi@cnr.it (B.S.); 2 CNR-SCITEC, Istituto di Scienze e Tecnologie Chimiche “Giulio Natta”, sezione di via Golgi, via C. Golgi 19, 20133 Milano, Italy; Carlo.Gatti@scitec.cnr.it; 3 Institute of Physical and Analytical Chemistry, School of Exact and Natural Sciences, Tbilisi State University, Chavchavadze Ave 3, 0179 Tbilisi, Georgia; jpba_bezhan@yahoo.com; 4 Strasbourg Institute of Chemistry, UMR CNRS 7177, Team LASYROC, 1 rue Blaise Pascal, University of Strasbourg, 67008 Strasbourg CEDEX, France; robin.weiss@unistra.fr (R.W.); ppale@unistra.fr (P.P.); 5 Crystallography, Magnetic Resonance and Modelling (CRM2), UMR CNRS 7036, University of Lorraine, Bd des Aiguillettes, 54506 Vandoeuvre-les-Nancy, France; emmanuel.aubert@univ-lorraine.fr; 6 Department of Molecular Sciences and Nanosystems DSMN, Venice Ca’ Foscari University, Via Torino 155, 30172 Mestre Venezia, Italy; cossu@unive.it

**Keywords:** bipyridines, chalcogen bond, electrostatic potential, enantioseparation, high-performance liquid chromatography, polysaccharide-based chiral stationary phases

## Abstract

The chalcogen bond (ChB) is a noncovalent interaction based on electrophilic features of regions of electron charge density depletion (σ-holes) located on bound atoms of group VI. The σ-holes of sulfur and heavy chalcogen atoms (Se, Te) (donors) can interact through their positive electrostatic potential (*V*) with nucleophilic partners such as lone pairs, π-clouds, and anions (acceptors). In the last few years, promising applications of ChBs in catalysis, crystal engineering, molecular biology, and supramolecular chemistry have been reported. Recently, we explored the high-performance liquid chromatography (HPLC) enantioseparation of fluorinated 3-arylthio-4,4′-bipyridines containing sulfur atoms as ChB donors. Following this study, herein we describe the comparative enantioseparation of three 5,5′-dibromo-2,2′-dichloro-3-selanyl-4,4′-bipyridines on polysaccharide-based chiral stationary phases (CSPs) aiming to understand function and potentialities of selenium σ-holes in the enantiodiscrimination process. The impact of the chalcogen substituent on enantioseparation was explored by using sulfur and non-chalcogen derivatives as reference substances for comparison. Our investigation also focused on the function of the perfluorinated aromatic ring as a π-hole donor recognition site. Thermodynamic quantities associated with the enantioseparation were derived from van’t Hoff plots and local electron charge density of specific molecular regions of the interacting partners were inspected in terms of calculated *V*. On this basis, by correlating theoretical data and experimental results, the participation of ChBs and π-hole bonds in the enantiodiscrimination process was reasonably confirmed.

## 1. Introduction

**Figure 1:**
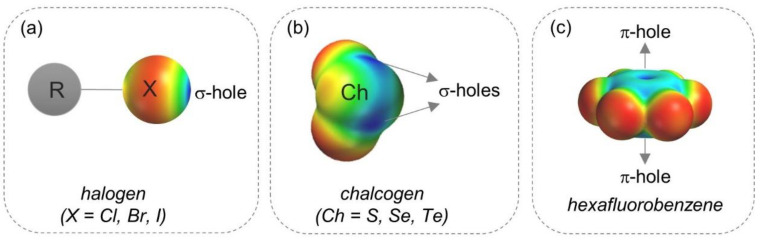
Location of halogen (**a**) and chalcogen (**b**) σ-holes and π-hole (**c**). For the *V*_S_ representations, colors towards red depict negative *V*_S_ while colors towards blue depict positive *V*_S_, and colors in between (orange, yellow, green) depict intermediate values.

The electronic charge distribution is anisotropic around bound atoms due to the rearrangement of electronic density when atoms participate in a bond formation, whereas free neutral atoms have spherically symmetric electronic density [1]. The terms σ-hole and π-hole describe the electronic charge density depletion which is observed in specific regions of bound atoms [2,3]. In most cases, positive electrostatic potential (*V*) values are associated with these regions [4]. Indeed, *V* in a point **r** (*V*(**r**)) is generated by each nucleus in a system and by the system’s electron distribution, given by Equation (1):

(1) $$V(r)\;=\;\sum_{A}\frac{Z_{A}}{\left|R_{A}-r\right|}-\int \frac{\rho \left(r\right)dr^{\prime }}{\left|r^{\prime }-r\right|}$$

where Z*_A_* is the positive charge on nucleus A located at *R_A_*, and *ρ*(**r**) is the electron density distribution [5,6]. Consequently, the sign of *V*(**r**) is positive if the effect of the nuclei (first positive term) is dominant due to a lower electron density at the point **r**. Otherwise, *V*(**r**) is negative if the effect of the electrons (second negative term) is dominant. σ-Holes were found on covalently bonded atoms of groups III–VIII and hydrogen, and depending on the atom valence, one or more σ-holes can be identified. In Figure 1, σ-and π-hole regions are depicted through *V* mapped on electron density isosurfaces (*V*_S_), colors toward red and blue representing negative and positive *V*_S_, respectively. σ-Hole regions on halogen (a) and chalcogen (b) atoms are located on the external side of the bound atom, approximately on the elongation of the covalent σ bond. Otherwise, an increase of electronic charge density occurs on the lateral sides of the atom. The σ-hole depth and the magnitude of the associated *V* depend on atom polarizability and electronegativity. The more polarizable and the less electronegative the atom is, the more positive the associated *V* may be. π-Hole regions are located above and below a planar portion of a molecule, often found on atoms involved in double bonds such as carbonyls and aromatic rings with strongly electron-withdrawing substituents. In Figure 1c, π-hole regions on hexafluorobenzene are represented. It is worth mentioning that *V* associated to σ- and π-hole is affected by the contributions from the entire molecule, and through-space effects may impact σ-hole depth in complex molecules [7,8,9]. Typically, electron-withdrawing groups covalently attached to an atom bearing σ- and/or π-hole redistributes the electronic density on the atom, increasing the depth of the “hole” and, consequently, its function as a Lewis acid. Neutral and cationic *N*-heterocyclic and perfluorinated substructures are able to activate σ-hole sites [10,11,12].

Their associated *V* being positive, σ- and π-hole regions (donors) can interact with negative sites (lone pairs, π-clouds, and anions) (acceptors). These types of noncovalent interactions are named σ- and π-hole bonds [1,13,14]. In this field, the most studied and applied interactions based on σ-hole are halogen (XB) [15] and chalcogen (ChB) [16] bonds involving atoms of groups VII (Cl, Br, I) and VI (S, Se, Te), respectively. In general, the interaction strength increases following the order Cl < Br < I and S < Se < Te. Currently, the importance of σ- and π-hole bonds has been recognized in several areas including catalysis, crystal engineering, molecular biology, molecular recognition, and supramolecular chemistry [17,18,19,20,21].

Despite the pivotal role of noncovalent interactions in chiral recognition, studies on the potentialities of σ- and π-hole bonds in enantioseparation science are scarce [22]. Starting from 2014, our groups demonstrated that (a) XB can work in HPLC environment [23,24,25], and (b) HPLC, as a technical tool, can be used to systematically investigate σ-hole bonds occurring on the surface of polysaccharide-based chiral stationary phases (CSPs) by properly tuning molecular properties of analyte as σ-hole donor and of selector as σ-hole acceptor, under normal phase (NP) elution conditions [26]. Recently, we reported the first investigation on ChB and π-hole bond in HPLC environment by using cellulose-based CSPs and fluorinated 3-arylthio-4,4′-bipyridines as analytes [27]. In these compounds, a sulfur atom at the 3-position was highly polarized by the electron-withdrawing 2,2′-dichloro-5,5′-dibromo-4,4′-bipyridine (**1**) framework.

**Figure 2:**
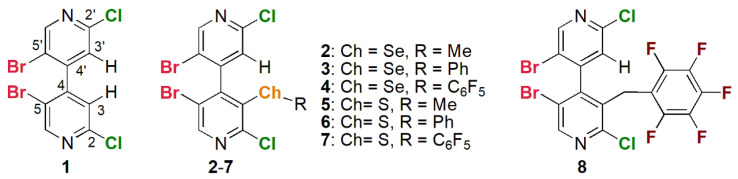
Structures of compounds **1**–**8**.

Following our previous studies, in this paper we describe the enantioseparation of 5,5′-dibromo-2,2′-dichloro-3-selanyl-4,4′-bipyridines **2**–**4** (Figure 2) on polysaccharide-based CSPs aiming to understand function and potentialities of selenium σ-holes in the enantiodiscrimination process. The impact of subtle structural variations on the chromatographic separation was also evaluated by comparing the chromatographic responses of **2**–**4** (3-SeR, R = Me, Ph, C_6_F_5_) and of compounds bearing different substituents at the 3-position of the 4,4′-bipyridine scaffold, namely **1** (3-H), 5,5′-dibromo-2,2′-dichloro-3-thio-4,4′-bipyridines **5**–**7** (3-SR, R = Me, Ph, C_6_F_5_), and the non-chalcogen analogue **8** (3-CH_2_R, R = C_6_F_5_).

In this study, the enantiomer elution order (EEO) was assigned, the effect of temperature was considered, and thermodynamic quantities associated with the enantioseparations were derived by van’t Hoff plots. Finally, calculated *V*_S_ values associated with σ- and π-hole regions of compounds **1**–**8** were correlated with the chromatographic parameters to disclose the potential role of ChBs and π-hole bonds in HPLC enantioseparation.

It is worth mentioning that selenium is an essential trace element which is present in selenoproteins with relevant biological functions [28]. Selenium compounds have become of interest for selenium supplementation for cancer chemoprevention [29]. In particular, selenomethionine is well known for its biological and dietary importance, the L-enantiomer being better absorbed into the body [30]. On this basis, enantioseparations of selenium and sulfur derivatives were reported in the literature, in general observing for selenium compounds higher retention and selectivity compared to sulfur analogues [31,32,33]. Nevertheless, the impact of σ-holes of sulfur and selenium on LC enantioseparation has been overlooked, and so far, ChB remains almost unexplored in enantioseparation science.

## 2. Results and Discussion

### 2.1. Conceptual Bases

The strength of ChB and π-hole interactions is regulated by the depth of the electron charge density hole, the Lewis basicity of the acceptor and the stereoelectronic properties of the medium. On the other hand, the pivotal components of an enantioseparation system are analyte, selector, and mobile phase (MP). Taking into account these features, HPLC can be used to investigate σ- and π-hole interactions occurring therein by properly tuning molecular properties of the analyte in this particular project as donor, selector as acceptor, and MP as medium. In particular, HPLC works in a solvated medium which can be easily tuned allowing solvent effects on both donor and acceptor to be evaluated. This approach was successfully applied to the study of XB in a HPLC environment [26]. Therefore, with the aim to demonstrate conclusively that ChBs and π-hole interactions can work in LC enantioseparations, an orthogonal screening was designed through a focused choice of analytes (ChB donors), CSPs (ChB acceptor), and MPs (ChB medium) considered as experimental variables. Changes of the chromatographic responses upon structural variations were evaluated in terms of changes in retention (*k*) and separation factors (α).

#### 2.1.1. Chiral Analytes

**Figure 3:**
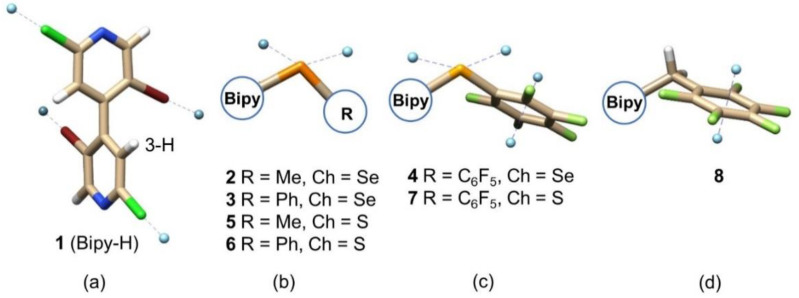
σ- (**a**–**c**) and π-hole (**c**,**d**) patterns in compounds **1**–**8**. Tube structure colors: bromine (red), chlorine (green), fluorine (clear green), hydrogen (white), nitrogen (blue), chalcogen (S, Se) (orange), σ-/π-hole (pale blue).

In this study, the identification and quantification of weak noncovalent interactions such as ChBs and π-hole bonds relies on the peculiar structure of the analytes used as test probes. Indeed, knowing that the enantioseparation of functionalized 4,4′-bipyridines depends on the substituents on the heteroaromatic scaffold [34,35], we expected that the chromatographic response would be strictly dependent on the σ- and π-hole of the distinctive substituent at the 3-position of compounds **1**1**–**8**8**. Recently, our groups designed and synthesized these compounds through focused procedures [11,27,36]. 4,4′-Bipyridines **1**–**8** are multi-site σ- and π-hole donors, the electron-withdrawing 4,4′-bipyridinyl moiety providing polarization of bound X and Ch atoms. Moreover, compounds **2**1**–**8**8** being chiral by atropisomerism, the absolute configuration of their pure enantiomers was assigned on the basis of X-ray diffraction (XRD) and electronic circular dichroism (ECD) analyses coupled with time-dependent density functional theory calculations (TD-DFT) [11,27]. Four different σ- and π-hole patterns could be identified in compounds **1**1**–**8**8** due to the features of the distinctive substituent at the 3-position (Figure 3): (a) compound **1** (Bipy-H) is the achiral 3-unsubstituted-4,4′-bipyridine containing four σ-holes located on the elongation of C_sp2_-Br and C_sp2_-Cl bonds; (b) compounds **2**, **3**, **5,** and **6** contain two additional σ-holes located on the elongation of Ch-Bipy and Ch-R bonds; (c) compounds **4** and **7** also contain a π-hole centered on the perfluorinated aromatic ring; (d) compound **8** contains a π-hole system but no Ch atom. Compounds **3**, **4**, and **7** were shown to function as σ- and π-hole donors. This was confirmed in the solid state through XRD analyses [9], and in solution through ^19^F NMR titration and catalysis experiments [11].

In previous studies, from two to four low-energy conformers were identified by calculation for compounds **2**–**8** [9,11] (Supplementary Data, Figure S1 and Table S1). Calculations of the *V*_S_ maximum (*V*_S,max_) values of the σ-holes showed that on average more positive *V*_S,max_ are associated with selenium σ-holes (compounds **2**–**4**) compared to sulfur (compounds **5**–**7**) due to the higher polarizability and lower electronegativity of selenium atom [9]. Moreover, the nature of R impacts the σ-hole depth which increases following the order Ph (**3**, **6**) < Me (**2**, **5**) < C_6_F_5_ (**4**,**7**) [9] (Supplementary Data, Table S1). The *V*_S,max_ on the π-hole increases in the order CH_2_C_6_F_5_ (**8**) < SeC_6_F_5_ (**4**) < SC_6_F_5_ (**7**). In each conformation, both σ- and π-holes are characterized by a distinctive chemical environment impacting the “hole” depth. Indeed, the impact of neighboring “holes” and through-space effects on the *V* associated with a σ- or a π-hole is particularly critical when the molecular system contains several “hole” sites. In this case, the contribution of a specific “hole” to the interaction capability of the molecular system may be challenging to be rationalized [37,38,39]. On the other hand, the study of multi-site σ-hole donors provides the possibility to examine in depth the actual capability of regions of electronic charge density depletion as interaction sites.

#### 2.1.2. Chiral Selectors

**Figure 4:**
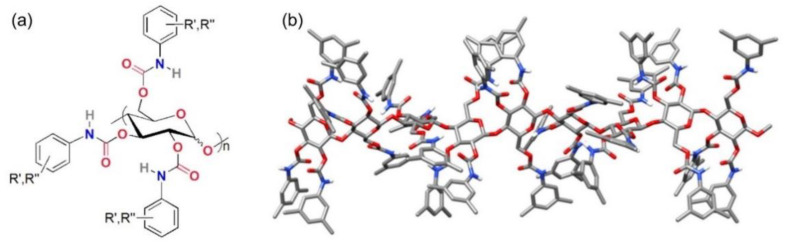
Drawing of polysaccharide carbamate-based selectors (**a**); three-dimensional (3D) tube structure of cellulose *tris*(3,5-dimethylphenylcarbamate) (**b**).

Polysaccharide phenylcarbamates, in addition to a presence of chiral centers, are characterized by conformational chirality dependent on the helical twist generated by the specific glycosidic 1,4-linkages in cellulose (β) and amylose (α) chains [40]. Carbamate groups are located deep inside the groove cavities near the polysaccharide backbone, while the hydrophobic aromatic rings tend to be located outside the chiral cavities (Figure 4). Thus, a chiral supramolecular environment surrounds the carbonyls potentially acting as ChB and/or π-hole acceptors, whereas the external aromatic rings are able to exert π–π and hydrophobic interactions. We recently demonstrated that XB-driven HPLC enantioseparations of halogenated 4,4′-bipyridines can be performed on cellulose *tris*(3,5-dimethylphenylcarbamate) (C-3,5diMe) [24,26], where XBs are formed between the halogen substituents of the analyte and the carbonyl groups of the CSP.

On this basis, two series of selectors (coated or immobilized on silica gel) were used in this study (Table 1), which contain carbonyls with different electron density distributions and, consequently, different capability as Lewis bases: (a) coated C-3,5diMe and amylose *tris*(3,5-dimethylphenylcarbamate) (A-3,5diMe) containing carbonyl groups with higher electron density due to the electron-donating effect induced by the methyl substituents at the 3- and 5-positions of the phenyl ring; (b) coated cellulose *tris*(3-chloro-4-methylphenylcarbamate) (C-3Cl,4Me) and amylose *tris*(5-chloro-2-methylphenylcarbamate) (A-5Cl,2Me), and the immobilized amylose *tris*(3-chloro-5-methylphenylcarbamate) (iA-3Cl,5Me) as chlorinated selectors, which present lower electron density on the carbonyl groups due to the electron-withdrawing effect exerted by the chlorine substituents of the phenyl ring.

The *V*_S_ minimum (*V*_S,min_) values calculated on the carbonyl oxygen atoms become less negative moving from methylated CSPs (−0.0660 au) to the chlorinated ones (−0.0618 au ≤ *V*_S,min_ ≤ −0.0594 au) [22]. In a complementary manner, the N-H amidic proton of the chlorinated CSPs has better capability as hydrogen bond (HB) donor (Lewis acid) than the methylated CSPs, and more positive *V*_S,max_ values were calculated in these cases [22]. In addition, the immobilized amylose *tris*(3,5-dimethylphenylcarbamate) (iA-3,5diMe) was also used to inspect the effect of immobilization on these series of enantioseparations.

The chromatographic responses of the polymeric selectors were compared under identical MPs in order to ensure a common environment assisting the interactions under investigation. If the analytes are able to exert ChBs as ChB donors on polysaccharides-3,5-diMe, lower retention and separation factors are expected on the chlorinated selectors due to the reduced capability of the carbonyls as “hole” acceptors.

#### 2.1.3. Mobile Phases

We used three MPs, namely *n*-hexane (Hex)/2-propanol (IPA) 90:10 (mix A), Hex/IPA/methanol (MeOH) 90:5:5 (mix B) and pure MeOH (mix C). ChB being based on the complementarity of donor and acceptor partners, the non-polar mix A was expected to assist the electrostatic interaction. Otherwise, MeOH-containing mixtures (mix B and mix C) were expected to destabilize the interactions by forming competitive HBs with the carbonyl oxygen atoms [24,41,42]. Meanwhile, MeOH favors hydrophobic contacts.

### 2.2. Chromatographic Screening

**Figure 5:**
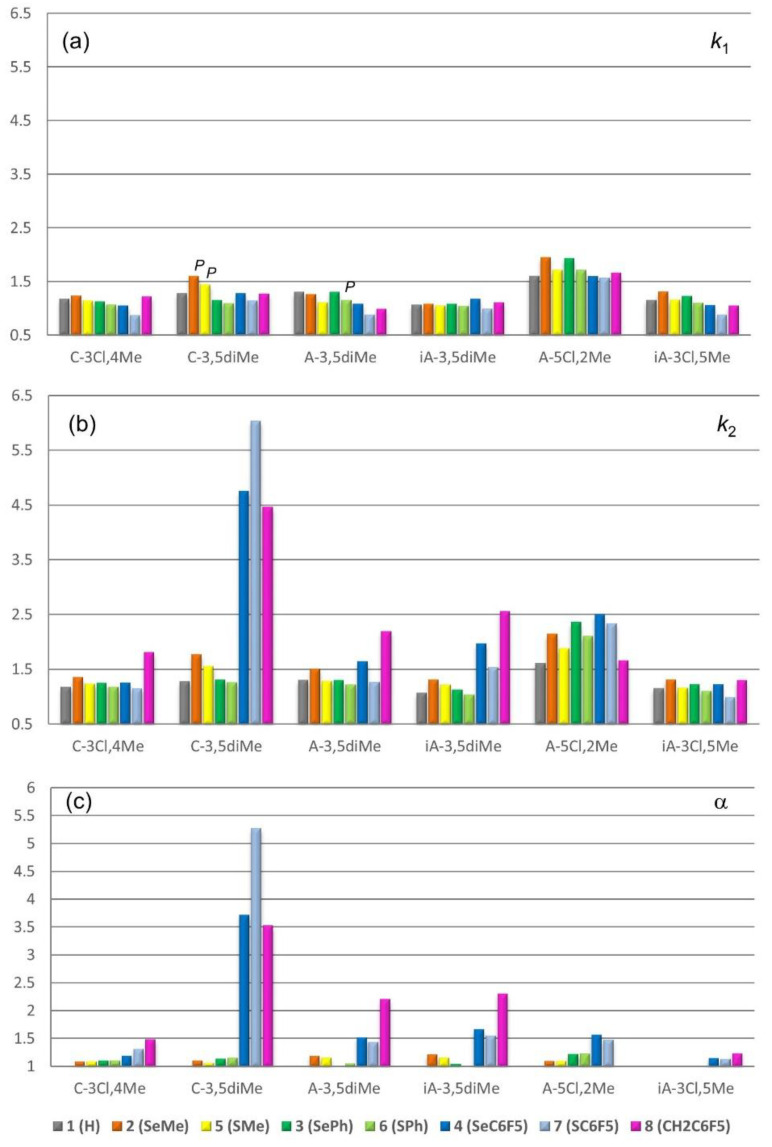
Retention of first (**a**) and second (**b**) eluting enantiomers and selectivity factors (**c**) of the enantioseparations of compounds **2**–**8** on seven polysaccharide-based CSPs. Compound **1** is used as a reference substance.

Retention factors of first (*k*_1_) and second (*k*_2_) eluted enantiomers, and selectivity factors (α) of the enantioseparations of compounds **2**–**8** on C-3,5diMe, C-3Cl,4Me, A-3,5diMe, iA-3,5diMe, A-5Cl,2Me, and iA-3Cl,5Me are summarized in Figure 5 (for numerical data see Supplementary Data, Table S2). On average, the selectivity factors increase following the order **3**, **6** < **5** < **2** < **4** < **8** < **7**. The best enantioseparations were obtained for compounds **3** and **6** (R = Ph) with A-5Cl,2Me (α = 1.22 and 1.23), for compounds **2** and **5** (R = Me) with the A-3,5diMe-based columns (α = 1.21 and 1.16) and for compounds **4**, **7,** and **8** (R = C_6_F_5_) with C-3,5diMe (α = 3.71, 5.27 and 3.52). This trend clearly showed the impact of the perfluorinated aromatic ring on the enantioseparation. This framework contains a π-hole region and exerts a strong electron-withdrawing effect on Ch and halogen atoms, increasing the depth of their σ-holes (Table S1). Retention was higher for selenium compounds **2**–**4** compared to the sulfur series (**5**–**7**) with all columns except one case with C-3,5diMe where **7** was more retained than **4**. The success rate of the CSPs in terms of average selectivity tended to increase as the *V*_S,min_ on the CSP carbonyl oxygen atoms decreases in the order iA-3Cl,5Me (average α, *V*_S,min_: 1.08, −0.0594 au), C-3Cl,4Me (1.20, −0.0606 au), A-5Cl,2Me (1.24, −0.0618 au), A-3,5diMe (1.37), iA-3,5diMe (1.42), C-3,5diMe (2.43) (−0.0660 au).

In general, retention, especially of the first eluting enantiomer, does not correlate with enantioresolution. Indeed, strong interactions occurring between analytes and CSP may not always contribute to the enantioselectivity. In this regard, scattered plots of ln α vs ln *k*_1_ were obtained for all CSPs with *p*-values ranging from 0.1009 to 0.7280, and no trend was observed. Otherwise, a tendency was found for the plots of ln α vs ln *k*_2_ of the three 3,5-dimethylated CSPs and the A-5Cl,2Me (0.0001 ≤ *p*-value ≤ 0.0146; 0.7280 ≤ *r*^2^ ≤ 0.9621) (Supplementary Data, Figure S2). In these cases, large *k*_2_ values were related to large separation factors (compounds **4**, **7,** and **8**), enantioseparation values increasing proportionally to retention. This observation indicated that improved enantioseparation is associated to stronger enantioselective interactions. No trend was observed for both plots of ln α vs ln *k*_1_ and ln α vs ln *k*_2_ in the case of C-3Cl,4Me and iA-3Cl,5Me. These columns have the best capability of the carbamate N-H as HB donor (*V*_S,max_ = 0.0868 and 0.0871 au, respectively). This result confirms that in most cases the analytes do not work as HB acceptors.

In fact, in the polysaccharide selectors, retention and selectivity are also determined by the conformational features of the polymer which are controlled by a series of intramolecular HBs between some of the carbonyl oxygen and the amidic hydrogen atoms [40,43]. In the chlorinated CSPs, as the electron density on both these HB centers changes due to the stereoelectronic effect of chlorine located on the aromatic rings, the strength of the HBs stabilizing the polymer conformation also changes impacting the conformation of the polymer.

Therefore, the unsuccessful trend which was observed with C-3Cl,4Me and iA-3Cl,5Me compared to C-3,5diMe and A-3,5diMe may be due to the change of polymer conformation, and thus not directly related to the lower capability of the carbonyl moieties of the CSPs as σ-hole acceptors. However, different behaviors could be observed. For the selanyl series **2**–**4** and the sulfur series **5**–**7** the reduction percentage of *k*_1_ and *k*_2_ increased in the order Ph < Me < C_6_F_5_ moving from C-3,5diMe to C-3Cl,4Me: SePh (**3**: −1.7%, −4.6%), SeMe (**2**: −22.5%, −23.7%), SeC_6_F_5_ (**4**: −18%, −73.5%) and SPh (**6**: −1.8%, −6.3%), SMe (**5**: −20.8%, −20%), SC_6_F_5_ (**7**: −23.7%, −80.9%). These values assumed an interesting meaning by considering that the average *V*_S,max_ on Ch (S, Se) σ-holes increased following the order Ph (0.0274, 0.0390 au) < Me (0.0348, 0.0441 au) < C_6_F_5_ (0.0470, 0.0553 au). For compound **8**, lacking Ch substituent, the reduction percentage of *k*_1_ and *k*_2_ changing from C-3,5diMe to C-3Cl,4Me (**8**: −3.9%, −59.4%) was lower compared to **4** and **7**. On this basis, in compounds **2**1**–**8**8**, the 3-substituents impacted *k*_2_ more than *k*_1_. This aspect was evident by comparing *k*_1_ and *k*_2_ of **2**1**–**8**8** with the retention of the achiral **1** as reference substance (Figure 5). The higher selectivity factors observed on the C-3,5diMe compared to the A-3,5diMe-based CSPs may be due to the wider chiral cavity of the cellulose-based polymer compared to the amylose-based system. Indeed, σ-hole bonds are interactions which require space, with typical C-Ch···acceptor angles around 180°.

The EEO was *M*-*P* in almost all cases. Reversal of the EEO dependent on backbone type was observed for compounds **2** and **5** (R = Me), showing *P*-*M* and *M*-*P* as the elution sequence on C-3,5diMe and A-3,5diMe, respectively. For the same pair of compounds, reversal of the elution sequence dependent on side chain structure was also observed, the EEO being *M*-*P* on C-3Cl,4Me. This effect based on the side chain structure was not observed in the amylose CSP series. Compound **6** also provided a case of EEO dependent on backbone structure, showing *M*-*P* and *P*-*M* as elution order on C-3,5diMe and A-3,5diMe, respectively. Interestingly, coated A-3,5diMe provided slight selectivity (α = 1.06) with *P*-*M* as EEO for compound **6** (Ch = S, R = Ph) but no enantioseparation for **3** (Ch = Se, R = Ph). Otherwise, compound **3** was enantioseparated with the opposite EEO (*M*-*P*) and similar selectivity (α = 1.05) on the immobilized iA-3,5diMe, which was unable to enantioseparate **6**.

### 2.3. Effect of Methanol on Retention and Selectivity

**Figure 6:**
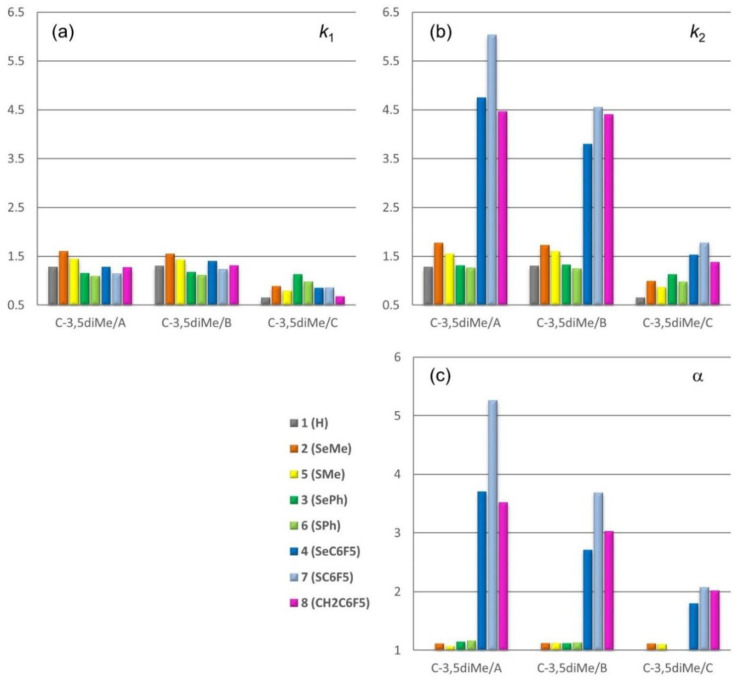
Retention of first (**a**) and second (**b**) eluted enantiomers and selectivity factors (**c**) of the enantioseparations of compounds **2**–**8** on C-3,5diMe with MP = Hex/IPA 90:10 *(mix A*), Hex/IPA/MeOH 90:5:5 (mix B), and pure MeOH (mix C). Compound **1** is used as a reference substance.

The impact of MP on retention and selectivity was evaluated by using mix B and C, containing different amount of MeOH, with C-3,5diMe. The results are summarized in Figure 6 and compared with the enantioseparation data obtained by using mix A on the same CSP (for numerical data see Supplementary Data, Table S3). Also in this case, scattered plots of ln α vs ln *k*_1_ were obtained for all CSPs with *p*-values ranging from 0.1171 to 0.9597, and no trend was observed. Otherwise, a tendency was found for the plots of ln α vs ln *k*_2_ (*p*-value = 0.0001 (mix B), 0.0072 (mix C); *r*^2^ = 0.9597 (mix B), 0.7931 (mix C)) (Supplementary Data, Figure S3). MP-dependent reversals of EEO were observed for compounds **2** and **5** (R = Me), the elution sequence being *P*-*M* and *M*-*P* with mix A and mix B/C, respectively.

The use of pure MeOH (mix C) as MP had a detrimental effect on the enantioseparation of **3** and **6** on C-3,5diMe, obtaining α = 1.00 in both cases. Enantioseparation of **2** remained unaffected in the polar medium, whereas α increased slightly for **5** (α = 1.07 (mix A) → 1.10 (mix C)). Otherwise, for compounds **4**, **7**, and **8**, the reduction percentages of k_2_ and α moving from mix A to mix C were higher with respect to the lowering observed for *k*_1_: **4** (*k*_1_, −33.6%; *k*_2_, −67.8%; α, −51.5%), **7** (*k*_1_, −24.6%; *k*_2_, −70.5%; α, −60.5%), and **8** (*k*_1_, −46.5%; *k*_2_, −69.0%; α, −33.5%). It is likely that, in this case, the higher hydrophobicity of selenium compared to sulfur lowers the reduction percentages observed for *k*_2_ and α of **4**.

The same trend could be observed with mix B, but with some exceptions. Indeed, for **4**, **7** and **8** *k*_2_ decreases (−20%, −24.5%, and −1.3%), whereas *k*_1_ increases (+ 9.4%, + 7.9%, and + 3.1%). This behavior highlights the impact of MeOH on the enantioseparation of both **4** and **7**, containing Ch substituents, compared to the non-chalcogen derivative **8**. In addition, these results also confirmed that retention of the first eluted enantiomers is determined by hydrophobic interactions, likely involving the four halogens on the 4,4′-bipyridine framework, whereas the retention of the second eluted enantiomer is controlled by the properties of the distinctive substituents at the 3-position. On the other hand, the retention of the reference compound **1** also increased with mix B (+ 1.5%) compared to mix A.

It is interesting to note that, analogously to XB behavior in HPLC environment [24], depending on substitution pattern and chromatographic medium (CSP structure and MP polarity), two competitive mechanisms, one hydrophobic and another involving “hole” regions, could be envisaged for the enantioseparation of **2**1**–**8**8** on polysaccharide-based CSPs. Considering compounds **4**, **7**, and **8,** due to their structural similarity, the hydrophobic mechanism provides very close values of *k*_2_ and α, whereas chromatographic parameters are spread over a wider range if the mechanism depends on the specific properties of either chalcogen substituents or π-hole. On this basis, *k*_2_ range becomes narrower moving from C-3,5diMe/mix A (0.29 ≤ Δ*k*_2_ ≤ 1.57) to C-3Cl,4Me/mix A (0.11 ≤ Δ*k*_2_ ≤ 0.66), C-3,5diMe/mix B (0.15 ≤ Δ*k*_2_ ≤ 0.75), and C-3,5diMe/mix C (0.15 ≤ Δ*k*_2_ ≤ 0.25). In particular, a drop of *k*_2_ was observed by replacing C-3,5diMe/mix A with C-3Cl,4Me/mix A (**4**: 4.75 → 1.26; **7**: 6.03 → 1.15; **8**: 4.46 → 1.81) due to the reduced ChB acceptor ability of the CSP (structural effect). Analogously, the polar interaction contribution to *k*_2_ was progressively suppressed on C-3,5diMe by changing the MP from mix A to mix B (**4**: 4.75 → 3.80; **7**: 6.03 → 4.55; **8**: 4.46 → 4.40) and mix C (**4**: 4.75 → 1.53; **7**: 6.03 → 1.78; **8**: 4.46 → 1.38) because alcohol exerts a cap-effect on the carbonyls of the CSP by means of competitive HB interactions (medium effect).

### 2.4. Effect of Temperature and Thermodynamic Quantities

The nature of the analyte/CSP contact can be explored on the basis of thermodynamic considerations. For each enantiomeric pairs, the difference in the change in standard enthalpy ΔΔH° and entropy ΔΔS° can be derived by the van’t Hoff equation (Equation (2)), taking into account that this approach does not differentiate between chiral and achiral contributions [44,45]. This equation describes the dependence between retention factor *k* and the absolute temperature T:

ln *k* = −ΔH°/*R*T + ΔS°/*R* + ln Φ (2)

where *R* is the gas constant and Φ is the phase ratio. ΔH° and ΔS° represent the differences in the enthalpy and entropy, respectively, when one enantiomer is adsorbed onto the CSP surface. Assuming that the plots of ln *k* against 1/T is linear in the temperature range of a study, the correlative thermodynamic parameters, which are temperature-independent, can be derived from the slope (ΔH° = −slope × *R*) and the intercept (ΔS* = intercept × *R*, where ΔS* is used to substitute the expression ΔS°/*R* + ln Φ) of the straight lines. The free energy associated with the adsorption of an enantiomer onto the CSP surface is given by the Gibbs–Helmholtz Equation (3):

ΔG° = ΔH° − T ΔS°(3)

ΔΔG°, ΔΔH°, and ΔΔS° represent the difference between the free energy of adsorption of the two enantiomers and its enthalpic and entropic terms, respectively (Equation (4)):

ΔΔG° = ΔΔH° − T ΔΔS°(4)

On the basis of the van’t Hoff equation (Equation (2)), ΔΔH° and ΔΔS° can be derived from Equation (5):

ln α = −ΔΔH°/*R*T + ΔΔS°/*R* (5)

Even if the molar quantities determined on the basis of Equation (2) are composite values representing different adsorption types, thermodynamic parameters are depending on analyte, CSP and MP, therefore useful information can emerge by comparison of thermodynamic data of analogous analyte/CSP pairs as subtle variations of the chromatographic system (analyte, CSP, MP) occur [46,47]. On this basis, retention and selectivity of compounds **2**–**8** were determined at different temperatures from 5 to 35 °C in 5 °C increments by using the following chromatographic systems (CSP/MP): C-3,5diMe/mix A, C-3Cl,4Me/mix A, C-3,5diMe/mix B, C-3,5diMe/mix C (Supplementary data, Tables S4–S7). From the evaluation of the thermodynamic quantities derived from van’t Hoff plots (Supplementary Data, Table S8), the following remarks emerged:

(i) enantioseparations were enthalpy-driven in all cases (|ΔΔH°| > |TΔΔS°|);
(ii) change in standard enthalpy and entropy were more negative on C-3,5diMe with mix A and mix B, indicating a stronger adsorption process under these conditions. A different trend was observed for compounds **2** and **5** (R = Me), showing more negative values with the system C-3,5diMe/mix C;
(iii) the ΔΔG° values associated with the enantioseparation of compounds **2** and **5** (R = Me) on the C-3,5diMe CSP showed to be quite different with mix A (ΔΔG° (kJ/mol) = −0.26, −0.19, respectively), whereas they became equal by using the same CSP with mix B (ΔΔG° = −0.27, −0.27), where methanol weakened analyte-CSP electrostatic interactions;
(iv) retention of both first and second eluted enantiomers were enthalpy-driven (|ΔH°| > |TΔS°|) in almost all cases. Entropy-driven retention (|ΔH°| < |TΔS°|) was observed for both enantiomers of compounds **2** and **5** (R = Me), and **6** (Ch = S, R = Ph) with the system C-3,5diMe/mix C. Under the same conditions, positive values of ΔG° were also derived for the first eluted enantiomers of compounds **4**, **7**, and **8**. The first eluted enantiomer of compound **7** gave entropy-driven retention also with the system C-3Cl,4Me/mix A. The adsorption step with positive ΔG° as an independent process is definitely impossible. However, it may be coupled with other endergonic processes facilitating the exergonic adsorption step. The details of this unusual observation are the subject of further studies;
(v) in all cases, thermodynamic quantities associated with retention changed in a narrower range (−1.17 ≤ ΔG° ≤ 0.96 kJ/mol) for the first eluted enantiomers compared to the second eluted ones (−4.45 ≤ ΔG° ≤ 0.36 kJ/mol), this evidence confirming that the adsorption mechanism of the most retained enantiomer is more sensitive to subtle structural variations;
(vi) in the series **4**–**7**–**8**, compound **7** showed the lowest retention for the first eluted enantiomer (ΔG° = −0.35 kJ/mol), and the highest retention for the second eluted enantiomer (ΔG° = −4.45 kJ/mol) with the system C-3,5diMe/mix A, evidencing the pivotal role of the system Ch = S and R = C_6_F_5_ for enantiodiscrimination.

### 2.5. Electrostatic Potential Analysis to Explore Chiral Recognition Mechanism

In previous studies, from two to four low-energy conformers were identified for compounds **2**–**8** [9,11] (Table S1 and Figure S1) by means of DFT calculations. These conformers originate from the relative orientation of the distinctive substituent at the 3-position, the methyl group (**2** and **5**), the phenyl (**3** and **6**), and the pentafluorophenyl (**4**, **7**, and **8**) rings. These substituents can be in front (conformers A) of the 2′-chloro-5′-bromo-4′-pyridyl ring or away from it (conformers B) due to rotation around the bond C3–Ch. For each of the two patterns A and B, two additional conformers are generated because the distinctive substituent at the 3-position can be close to the 3′-hydrogen (conformers A1 and B1) or to the 5′-bromine atom (conformers A2 and B2). On this basis, conformers **2**-A2, **3**-A1, **4**-B1, **5**-A2, **6**-A1, **7**-B1, and **8**-B1 were calculated as the most stable in vacuum [9]. In this regard, it is worth noting that A2-type conformers were found as the most populated in vacuum only for compounds **2** and **5**, whereas 1-type conformers (A1 and B1) were computed as most stable for the other compounds. On this basis, the different position of the methyl group with respect to the 4,4′-bipyridine framework could explain the reversal of EEO observed with C-3,5diMe and mix A. As *V*_S,max_ values of X and Ch σ-holes and phenyl π-holes have been computed for each conformers (Table S1) [9], we have applied here *V* analysis of analytes and polysaccharide-based CSPs, recently used to successfully rationalize noncovalent interaction patterns underlying enantiodiscrimination [47,48].

Considering compounds **4**, **7**, and **8**, both retention and selectivity obtained with C-3,5diMe and mix A increase in the order **8** (4.46, 3.52) < **4** (4.75, 3.71) < **7** (6.03, 5.27) (Table S2) as the *V*_S,max_ values calculated for the π-hole regions of conformers B1 (*V*_S,max_ [au], **8**: 0.0369, 0.0416 < **4**: 0.0455, 0.0425 < **7**: 0.0470, 0.0444), indicating that the π-hole region participates in the enantiodiscrimination process as electrophilic site.

**Figure 7:**
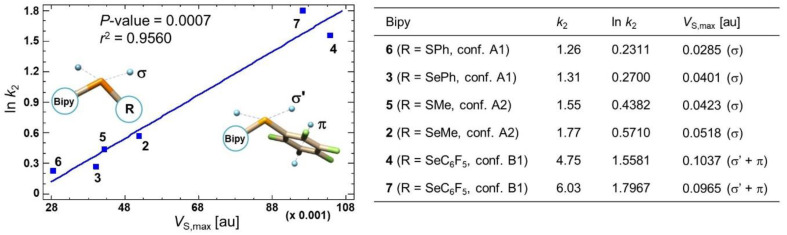
Linear regression analysis describing the relationships between ln *k*_2_ (C-3,5diMe, Hex/IPA 90:10) and *V*_S,max_ values for compounds **2**–**7**.

With the aim to also confirm the participation of the Ch σ-hole in the enantiodiscrimination processes, the possible correlation between calculated *V*_S,max_ values and retention of the second eluted enantiomers of compounds **2**–**7** with C-3,5diMe and mix A was explored by fitting a simple linear regression model to describe the relationship between ln *k*_2_ and *V*_S,max_ as independent variable. Good correlation (*p*-value = 0.0007, *r*^2^ = 0.9560) (Figure 7) was obtained by considering the *V*_S,max_ values associated on the σ-hole located on the elongation of the C_pyridyl_-Ch bond for compounds **2**, **3**, **5**, and **6**. Otherwise, the sum of this σ-hole and the π-hole region was considered for compounds **7** and **4**. It is worth noting that it was reasonable to neglect the σ-hole located on the elongation of the C_R_–Ch bond as well as the π-hole below the aromatic plane, both regions being less accessible.

It is worth mentioning that good correlation levels were also obtained with a larger number of inspected compounds by fitting a simple linear regression model to describe the relationships between ln *k*_2_ vs *V*_S,max_ (C_pyridyl_-Ch σ-hole) and ln *k*_2_ vs *V*_S,max_ (Ar π-hole), as independent variables (Supplementary Data Figure S4). The Ch σ-holes of compounds **2**–**7** and the fluoroaryl derivatives **9**–**14** [27] (n. 12), and the π-holes of compounds **3**, **4**, **6**–**8**, and **11**1**–**8**14** (n. 9) were considered. In both cases, *p*-values < 0.05 were obtained indicating a statistically significant correlation for the examined relationships: ln *k*_2_ = *f(V*_S,max_ (C_pyridyl_-Ch σ-hole)), *p*-value = 0.0033, *r*^2^ = 0.5946; ln *k*_2_ = *f(V*_S,max_ (Ar π-hole)), *p*-value = 0.0001, *r*^2^ = 0.9146.

### 2.6. Source Function Reconstruction of the Electrostatic Potential

Given *V*_S,max_ associated with a “hole” region, this *V* value may be envisaged as being due to source contributions from atoms or groups of the system. The decomposition of *V* in atomic groups contributions can be achieved by extending the Bader–Gatti source function (SF) for the electron density to *V* [9,27,49,50] in order to quantify the impact of single contributions to the *V* value. On this basis, the factors determining a certain *V* in a point can be inspected, understanding the reasons of observed trends in series of structurally related compounds.

**Figure 8:**
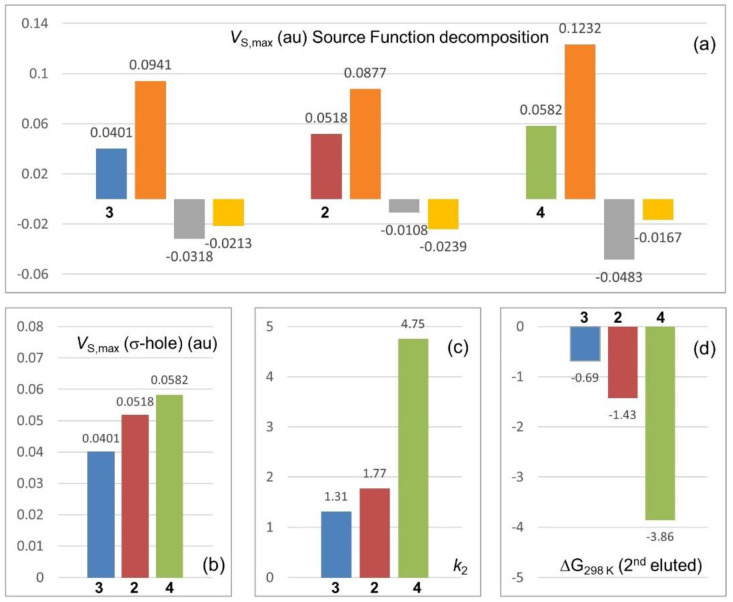
(**a**) *V*_S,max_ (au) source function (SF) decomposition in three σ-hole cases (compounds **2**, **3**, and **4**) [9], data refer to the C_pyridyl_–Se σ-holes, contribution colors: Se (orange), R (grey), Bipy (yellow); (**b**) *V*_S,max_ (au) associated with the C_pyridyl_–Se σ-holes of compounds **2**–**4**, (**c**) trend of *k*_2_, and (**d**) ΔG°_298 K_ for the second eluted enantiomers of compounds **2**–**4**.

The results of the SF decomposition of *V*_S,max_ on the basis of the equation *V*_S,max_ = SF(Se) + SF(R) + SF(Bipy), calculated for the C_pyridyl_-Se σ-hole of selenium compounds **2**, **3**, and **4**, are reported in Figure 8a. The *V*_S,max_ (Figure 8a, leftmost bar for each compound) originates from the sum of single contributions of each component of the molecular system, namely the Se atom itself (Figure 8, orange), the 4,4′-bipyridine framework (yellow), and the distinctive substituent R (grey). The source sign is positive or negative whether the atomic (or group) source concurs or opposes to the positive potential of the σ-hole.

In the case of **2**, the methyl group is not really “electron-withdrawing” but the σ-hole depth is not negligible (*V*_S,max_ = 0.0518 au) due to the less negative SF contribution of the methyl group (−0.0108 au) to the C_pyridyl_-Se σ-hole. In the case of **3**, the higher electron-withdrawing power of the phenyl relative to the methyl group increases the positive SF contribution of selenium compared to **2** (0.0877, **2** → 0.0941, **3**), but a more negative contribution from the Ph group (−0.0108, **2** → −0.0318, **3**) makes the *V*_S,max_ less positive (0.0401 au). Finally, for compound **4**, the negative contribution of the C_6_F_5_ group increases (−0.0483 au), but it is balanced by a less negative contribution of the bipyridine framework (−0.0167 au) and the strong positive contribution of the Se itself (+ 0.1232 au), due to the very high electron-withdrawing power of the C_6_F_5_ group. The SF reconstruction provides a theoretical explanation of *V*_S,max_ origin and trend observed for compounds **2**–**4** (Figure 8b). In turn, the retention features of the second eluted enantiomers of compounds **2**–**4** (Figure 8c,d) change coherently with *V*_S,max_ on the C_pyridyl_-Se σ-hole confirming the participation of this region to the enantiodiscrimination process.

## 3. Conclusions

In most cases, so far sulfur and selenium sites have been considered to participate in LC retention and enantiodiscrimination as weak HB acceptor and hydrophobic site, respectively. However, if properly activated by the electron-withdrawing properties of R and R’ frameworks, sulfur and selenium compounds with general formula R–S–R’ and R-Se-R’ present two σ-holes regions located on the elongation of the σ bonds R-Ch and R’-Ch (Ch = S, Se). Analogously, perfluorinated aromatic rings contain regions of electron charge density depletion (π-hole) located above and below the molecular plane. Our study reasonably confirmed that noncovalent interactions involving these regions can participate in both retention and enantioseparation mechanisms. In the last few years, new achievements in the theoretical knowledge shed light on the nature and functions of σ- and π-holes in several fields, introducing a deep change in the way research looks at the chalcogen bond utilization. In this perspective, our exploration paves the way for repositioning chalcogen sites also in enantioseparation science.

## 4. Materials and Methods

### 4.1. Chemistry

Compounds **1** and r*ac*-**2**–**8** were synthesized as previously reported [11,27,36]. HPLC-grade *n*-hexane (Hex), methanol (MeOH), and 2-propanol (IPA) were purchased from Sigma-Aldrich (Taufkirchen, Germany).

### 4.2. Chromatography

An Agilent Technologies (Waldbronn, Germany) 1100 Series HPLC system (high-pressure binary gradient system equipped with a diode-array detector operating at multiple wavelengths (220, 254, 280, 360 nm), a programmable autosampler with a 20 μL loop, and a thermostated column compartment) was employed. Data acquisition and analyses were carried out with Agilent Technologies ChemStation Version B.04.03 chromatographic data software. The UV absorbance is reported as milliabsorbance units (mAU). Lux Cellulose-1 (cellulose *tris*(3,5-dimethylphenylcarbamate)), Lux Cellulose-2 (cellulose *tris*(3-chloro-4-methylphenylcarbamate)), Lux Amylose-1 and Lux i-Amylose-1 (amylose *tris*(3,5-dimethylphenylcarbamate)), Lux Amylose-2 (amylose *tris*(5-chloro-2-methylphenylcarbamate)), and Lux i-Amylose-3 (amylose *tris*(3-chloro-5-methylphenylcarbamate)) (5 μm) (Phenomenex Inc., Torrance, CA, USA) were used as chiral columns (250 × 4.6 mm). The retention factor (*k*) was determined as *k* = (*t*_R_-*t*_0_)/*t*_0_, where *t*_R_ is the retention time for the eluted enantiomer. Dead time (*t*_0_) was measured by injection of *tri*-*tert*-butylbenzene (Sigma-Aldrich, Taufkirchen, Germany) as a non-retained compound [51]. The separation factor (α) was calculated as α = *k*_2_/*k*_1_, where *k*_1_ and *k*_2_ are the retention factors of the first- and the second-eluted enantiomer, respectively. Analyses were performed in isocratic mode at 25 °C. The flow rate (FR) was set at 0.8 mL/min. For compounds **2**–**8**, the enantiomer elution order (EEO) was determined by injecting enantiomers of known absolute configuration [11,27]. The van’t Hoff experiments were conducted at 5, 10, 15, 20, 25, 30, and 35 °C by using a thermostat jacket equipped with a RE104 LAUDA circulating water-bath (Lauda, Königshofen, Germany) (resolution 0.1 °C; accuracy ± 0.4 °C; temperature control ± 0.02 °C). When the temperature was changed, the column was allowed to equilibrate for 1 h before injecting the samples. Thermodynamic parameters were derived from the slopes and the intercepts of the van’t Hoff plots by linear regression analysis. Statgraphics Centurion XVI (Statpoint Technologies, Inc., Warrenton, VA, USA) was used for all linear regression analyses.

### 4.3. Computationals

Electrostatic potential extrema on the molecular electron density isosurfaces (maxima and minima) (*V*_S,max_ and *V*_S,min_) were calculated as previously reported at DFT/B3LYP/6-311G* level [9] by using Gaussian 09 (Wallingford, CT, USA) [52], and given in au (electrons/bohr). Search for the exact location of *V*_S,max_ and *V*_S,min_ was made through the Multiwfn code [53] and through its module enabling quantitative analyses of molecular surfaces (isovalue 0.002 au) [54]. The SF reconstruction of *V*_S,max_ was performed as previously described [9,27].

## Figures and Tables

**Table 1:** Structure of the six polysaccharide-based chiral stationary phases (CSPs) used in the study.

Column ^1^	Backbone	Ar (R’,R’’-C_6_H_4_)	Abbreviation	*V*_S,min C=O_ (au) ^2^	*V*_S,max N-H_ (au) ^2^
Lux Cellulose-1	Cellulose	3,5-dimethyl	C-3,5diMe	−0.0660	0.0788
Lux Cellulose-2	Cellulose	3-chloro-4-methyl	C-3Cl,4Me	−0.0606	0.0868
Lux Amylose-1	Amylose	3,5-dimethyl	A-3,5diMe	−0.0660	0.0788
Lux i-Amylose-1	Amylose	3,5-dimethyl	iA-3,5diMe	−0.0660	0.0788
Lux Amylose-2	Amylose	5-chloro-2-methyl	A-5Cl,2Me	−0.0618	0.0798
Lux i-Amylose-3	Amylose	3-chloro-5-methyl	iA-3Cl,5Me	−0.0594	0.0871

^1^ Coated columns: Lux Cellulose-1, Cellulose-2, Amylose-1, Amylose-2. Immobilized columns: Lux i-Amylose-1 and i-Amylose-3. ^2^ *V*_S_ values calculated at DFT/B3LYP/6-311G* level [22].

## Data Availability

Original HPLC chromatograms and details of computed structures are available on request from the corresponding author.

## Supplementary Materials

The following are available online: Figure S1: Conformations and related *V*_S_ representations on electron density isosurfaces of compounds **2**–**8**, Table S1: *V*_S,max_ (au) on halogen (Cl, Br), sulfur and selenium σ-holes (0.002 au), and the pentafluorophenyl ring π-hole calculated for conformers of compounds **1**–**8**, Table S2: Retention and selectivity of 4,4′-bipyridines **1**1**–**8**8** on coated and immobilized cellulose- and amylose-based CSPs by using Hex/IPA 90:10 as MP, Table S3: Retention and selectivity of 4,4′-bipyridines **1**–**8** on coated Lux Cellulose-1 (C-3,5diMe) by using Hex/IPA 90:10 (mix A), Hex/IPA/MeOH 90:5:5 (mix B) and MeOH (mix C) as mobile phases, Figure S2: Linear regression analysis describing the relationships between ln α and ln k_2_ for **2**1**–**8**8** on cellulose- and amylose based CSPs, Figure S3: Linear regression analysis describing the relationships between ln α and ln *k*_2_ for **2**–**8** on C-3,5diMe with mix B and mix C as MPs, Table S4: Temperature dependence of retention factors and van’t Hoff equations for 4,4′-bipyridines **2**–**8** on C-3,5diMe (Lux Cellulose-1), hex/IPA 90:10 (mix A), Table S5: Temperature dependence of retention factors and van’t Hoff equations for 4,4′-bipyridines **2**–**8** on C-3Cl,4Me (Lux Cellulose-2), hex/IPA 90:10 (mix A), Table S6: Temperature dependence of retention factors and van’t Hoff equations for 4,4′-bipyridines **2**–**8** on C-3,5diMe (Lux Cellulose-1), hex/IPA/MeOH 90:5:5 (mix B), Table S7: Temperature dependence of retention factors and van’t Hoff equations for 4,4′-bipyridines **2**1**–**8**8** on C-3,5diMe (Lux Cellulose-1), MeOH 100% (mix C), Table S8: Thermodynamic quantities calculated from the van’t Hoff plots for 4,4′-bipyridines **2**–**8** on C-3,5diMe and C-3Cl,4Me under normal phase (NP) an polar organic (PO) elution modes, Figure S4: Linear regression analysis describing the relationships between ln *k*_2_ (C-3,5diMe, mix A) and *V*_S,max_ (C_pyridyl_-Ch σ-hole) (au) (compounds **2**1**–**8**7** and **9**1**–**8**14**) (a) and *V*_S,max_ (Ar π-hole) (au) (compounds **3**, **4**, **6**–**8**, and **11**–**14** (b).
