# Supplementary material for: Enantioseparation of 5,5′-Dibromo-2,2′-dichloro-3-selanyl-4,4′-bipyridines on Polysaccharide-Based Chiral Stationary Phases: Exploring Chalcogen Bonds in Liquid-Phase Chromatography
Source: Molecules. 2021 Jan 4;26(1):221. doi: 10.3390/molecules26010221 (PMC7794968; doi:10.3390/molecules26010221)
Supplement: Supplementary file 1 [file molecules-26-00221-s001.pdf]

Supplementary data

Enantioseparation of 5,5′-dibromo-2,2′-dichloro-3-selanyl-4,4′-bipyridines on polysaccharide-based chiral stationary phases: exploring chalcogen bonds in liquid-phase chromatography

Paola Peluso [1,*], Alessandro Dessì [1], Roberto Dallocchio [1], Barbara Sechi [1], Carlo Gatti [2], Bezhan Chankvetadze [3], Victor Mamane [4,*], Robin Weiss [4], Patrick Pale [4], Emmanuel Aubert [5], and Sergio Cossu [6]

[1] Institute of Biomolecular Chemistry ICB, CNR, Secondary branch of Sassari, Traversa La Crucca 3, Regione Baldinca, Li Punti, 07100 Sassari, Italy; alessandro.dessi@cnr.it (A.D.); roberto.dallocchio@cnr.it (R.D.); barbara.sechi@cnr.it (B.S.)

[2] CNR-SCITEC, Istituto di Scienze e Tecnologie Chimiche "Giulio Natta", sezione di via Golgi, via C. Golgi 19, 20133 Milano, Italy; Carlo.Gatti@scitec.cnr.it (C.G.)

[3] Institute of Physical and Analytical Chemistry, School of Exact and Natural Sciences, Tbilisi State University, Chavchavadze Ave 3, 0179 Tbilisi, Georgia; jpba_bezhan@yahoo.com (B.C.)

[4] Strasbourg Institute of Chemistry, UMR CNRS 7177, Team LASYROC, 1 rue Blaise Pascal, University of Strasbourg, 67008 Strasbourg CEDEX, France; robin.weiss@unistra.fr (R.W.); ppale@unistra.fr (P.P.)

[5] Crystallography, Magnetic Resonance and Modelling (CRM2), UMR CNRS 7036, University of Lorraine, Bd des Aiguillettes, 54506 Vandoeuvre-les-Nancy, France; emmanuel.aubert@univ-lorraine.fr (E.A.)

[6] Department of Molecular Science and Nanosystems DSMN, Venice Ca' Foscari University, Via Torino 155, 30172 Mestre Venezia, Italy; cossu@unive.it (S.C.)

* Correspondence: paola.peluso@cnr.it (P. Peluso); vmamane@unistra.fr (V.M.); Tel.: +39-079-2841218 (P. Peluso); +33-3-68851612 (V.M.)

Table of contents

		pag.
**Figure S1.**	Conformations calculated for compounds **2**-**8** and V_S representations on electron density isosurfaces	3
**Table S1.**	$V_{S,max}$ on halogen (Cl, Br), sulfur and selenium σ-holes, and the pentafluorophenyl ring π-hole calculated for conformers of compounds **1**-**8**	4
**Table S2.**	Retention and selectivity of 4,4′-bipyridines **1**-**8** on coated and immobilized cellulose- and amylose-based CSPs by using Hex/IPA 90:10 as mobile phase	5
**Table S3.**	Retention and selectivity of 4,4′-bipyridines **1**-**8** on coated Lux Cellulose-1 (C-3,5diMe) by using Hex/IPA 90:10 (*mix A*), Hex/IPA/MeOH 90:5:5 (*mix B*) and MeOH (*mix C*) as mobile phases	5
**Figure S2.**	Linear regression analysis describing the relationships between ln α and ln k_2 for **2**-**8** on cellulose- and amylose based CSPs	6
**Figure S3.**	Linear regression analysis describing the relationships between ln α and ln k_2 for **2**-**8** on C-3,5diMe with *mix B* and *mix C* as MPs	6
**Table S4.**	Temperature dependence of retention factors and van't Hoff equations for 4,4′-bipyridines **2**-**8** on C-3,5diMe (Lux Cellulose-1), hex/IPA 90:10 (*mix A)*	7
**Table S5.**	Temperature dependence of retention factors and van't Hoff equations for 4,4′-bipyridines **2**-**8** on C-3Cl,4Me (Lux Cellulose-2), hex/IPA 90:10 (*mix A)*	8
**Table S6.**	Temperature dependence of retention factors and van't Hoff equations for 4,4′-bipyridines **2**-**8** on C-3,5diMe (Lux Cellulose-1), hex/IPA/MeOH 90:10 (*mix B)*	9
**Table S7.**	Temperature dependence of retention factors and van't Hoff equations for 4,4′-bipyridines **2**-**8** on C-3,5diMe (Lux Cellulose-1), MeOH 100% (*mix C)*	10
**Table S8.**	Thermodynamic quantities calculated from the van't Hoff plots for 4,4′-bipyridines **2**-**8** on C-3,5-diMe and C-3Cl,4Me under normal phase (NP) and polar organic (PO) elution modes	11
**Figure S4.**	Linear regression analysis describing the relationships between ln k_2 (C-3,5diMe, *mix A*) and $V_{S,max}$ ($C_{pyridyl}$-Ch σ-hole) [au] (compounds **2**-**7** and **9**-**14**) (a) and $V_{S,max}$ (Ar π-hole) [au] (compounds **3**, **4**, **6**-**8**, and **11**-**14**) (b)	12

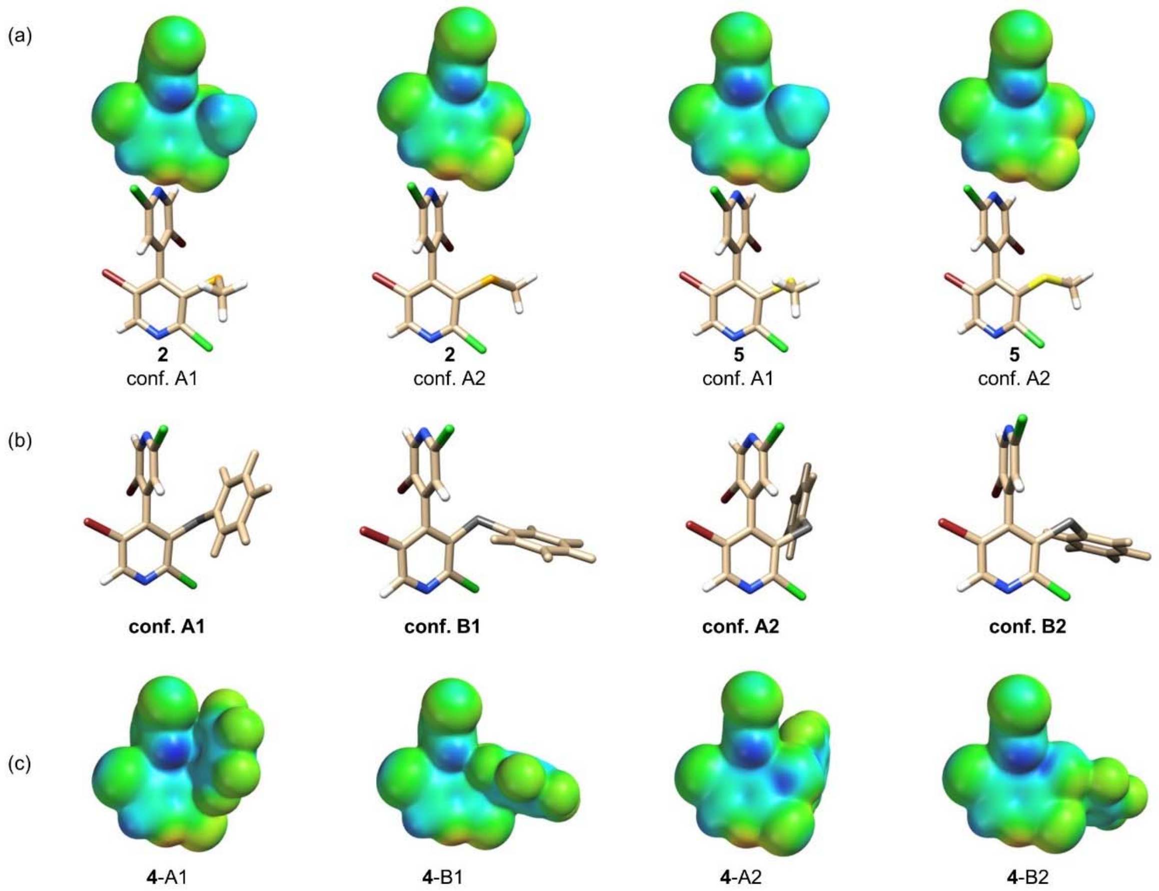

Figure S1. Conformations and related V_S representations on electron density isosurfaces (0.002 au) graphically generated by using Spartan' 10 (DFT/B3LYP/6-311G*) [1S]: (a) V_S representations and tube structures of conformers A1 and A2 calculated for compound **2** (Ch = Se) and **5** (Ch = S) (R = Me), (b) conformation motifs A1, B1, A2 and B2 computed for compounds **4**, and **6-8** (for compound **3** only conformers A1 and A2 were identified by calculations), and (c) V_S representations of conformers A1, B1, A2, and B2 of compound **4**. Tube structures colours: bromine (red), chalcogen S/Se (dark grey), chlorine (green), hydrogen (white), nitrogen (blue), selenium (orange), sulfur (yellow). For the V_S representations, colours towards red depict negative V_S, while colours towards blue depict positive V_S, and colours in between (orange, yellow, green) depict intermediate values.

[1S] Shao, Y.; Molnar, L.F.; Jung, Y.; Kussmann, J.; Ochsenfeld, C.; Brown, S.T.; Gilbert, A.T.B.; Slipchenko, L.V.; Levchenko, S.V.; O'Neil, D.P.; Di Stasio Jr, R.A.; Lochan, R.C.; Wang, T.; Beran, G.J.O.; Besley, N.A.; Herbert, J.M.; Lin, C.Y.; VanVoorhis, T.; Chien, S.H.; Sodt, A.; Steele, R.P.; Rassolov, V.A.; Maslen, P.E.; Korambath, P.P; Adamson, R.D.; Austin, B.; Baker, J.; Byrd, E.F.C.; Dachsel, H.; Doerksen, R.J.; Dreuw, A.; Dunietz, B.D.; Dutoi, A.D.; Furlani, T.R.; Gwaltney, S.R.; Heyden, A.; Hirata, S.; Hsu, C.-P.; Kedziora, G.; Khalliulin, R.Z.; Klunzinger, P.; Lee, A.M.; Lee, M.S.; Liang, W.Z.; Lotan, I.; Nair, N.; Peters, B.; Proynov, E.I.; Pieniazek, P.A.; Rhee, Y.M.; Ritchie, J.; Rosta, E.; Sherrill, C.D.; Simmonett, A.C.; Subotnik, J.E.; Woodcock III, H.L.; Zhang, W.; Bell, A.T.; Chakraborty, A.K.; Chipman, D.M.; Keil, F.J.; Warshel, A.; Hehre, W.J.; Schaefer, H.F.; Kong, J.; Krylov, A.I.; Gill, P.M.W.; Head-Gordon, M. Advances in methods and algorithms in a modern quantum chemistry program package. *Phys. Chem. Chem. Phys.* **2006**, *8*, 3172–3191 (DOI: 10.1039/b517914a).

Table S1. $V_{S,max}$ [au] on halogen (Cl, Br), sulfur and selenium σ-holes (0.002 au), and the pentafluorophenyl ring π-hole calculated for conformers of compounds **1**-**8** (substituent at the 3-position 3-YR, with Y = S, Se, CH_2 and R = Me, Ph, C_6F_5) (B3LYP/6-311G*).[1]

Conf.	R	Y	2′-Cl	2-Cl	5′-Br	5-Br	Ch (σ-hole $C_{pyridyl}$-Ch)	Ch (σ-hole C_R-Ch)	π-hole external[2]	π-hole internal[2]
**1**	H	--	0.0248	0.0248	0.0484	0.0484	--	--	--	--
**2**-A1	Me	Se	0.0229	0.0253	0.0452	0.0471	0.0521	0.0260		
**2**-A2			0.0219	0.0256	0.0486	0.0470	0.0518	0.0463		
**3**-A1	Ph	Se	0.0225	0.0224	0.0443	0.0466	0.0401	0.0352	-0.0072	--
**3**-A2			0.0212	0.0225	0.0441	0.0463	0.0391	0.0414	-0.0082	-0.0147
**4**-A1	C_6F_5	Se	0.0251	0.0266	0.0493	0.0514	0.0584	0.0559	0.0454	0.0527
**4**-A2			0.0244	0.0267	0.0512	0.0512	0.0577	0.0609	0.0448	--
**4**-B1			0.0235	0.0294	0.0496	0.0510	0.0582	0.0319[3]	0.0455	0.0425
**4**-B2			0.0258	0.0314	0.0476	0.0510	0.0593	0.0603	0.0438	0.0390
**5**-A1	Me	S	0.0233	0.0259	0.0449	0.0478	0.0428	0.0193		
**5**-A2			0.0219	0.0262	0.0493	0.0476	0.0423	--		
**6**-A1	Ph	S	0.0224	0.0230	0.0445	0.0475	0.0285	0.0268	-0.0080	--
**6**-A2			0.0215	0.0231	0.0451	0.0471	0.0267	0.0340	-0.0090	-0.0138
**6**-B1			0.0221	0.0222	0.0446	0.0469	0.0307	0.0169	-0.0115	-0.0087
**6**-B2			0.0215	0.0220	0.0491	0.0464	0.0286	--	-0.0127	-0.0093
**7**-A1	C_6F_5	S	0.0258	0.0269	0.0520	0.0492	0.0491	0.0445	0.0466	0.0538
**7**-A2			0.0246	0.0271	0.0519	0.0518	0.0491	0.0501	0.0456	--
**7**-B1			0.0235	0.0307	0.0486	0.0512	0.0495	0.0272[4]	0.0470	0.0444
**7**-B2			0.0250	0.0326	0.0475	0.0512	0.0512	0.0553	0.0452	0.0404
**8**-A1	C_6F_5	CH_2	0.0256	0.0271	0.0511	0.0491	--	--	0.0418	0.0459
**8**-A2			0.0259	0.0274	0.0502	0.0489	--	--	0.0409	--
**8**-B1			0.0252	0.0277	0.0509	0.0488	--	--	0.0369	0.0416
**8**-B2			0.0273	0.0287	0.0479	0.0490	--	--	0.0340	0.0398

[1] V_S values for compounds **2**-**8** from Ref. [9]. [2] External and internal π-holes are oriented far from and close to the 4,4′-bipyridine moiety, respectively. [3] A third σ-hole on the elongation of the C_{ArF}-Se was found for the conformer **4**-B1 with $V_{S,max}$ = 0.0309 au. [4] A third σ-hole on the elongation of the C_{ArF}-S was found for the conformer **7**-B1 with $V_{S,max}$ = 0.0202 au.

Table S2. Retention and selectivity of 4,4′-bipyridines **1**-**8** on coated and immobilized cellulose- and amylose-based CSPs by using Hex/IPA 90:10 as mobile phase (*FR* 0.8 ml/min, T = 25°C) (the absolute configuration of eluted enantiomers is reported in brackets).

CSP[1]	k_1, k_2, α	**1**	**2**	**3**	**4**	**5**	**6**	**7**	**8**
C-3,5diMe	k_1	1.28	1.60 (*P*)	1.15 (*M*)	1.28 (*M*)	1.44 (*P*)	1.09 (*M*)	1.14 (*M*)	1.27 (*M*)
	k_2		1.77 (*M*)	1.31 (*P*)	4.75 (*P*)	1.55(*M*)	1.26 (*P*)	6.03 (*P*)	4.46 (*P*)
	α		1.11	1.14	3.71	1.07	1.16	5.27	3.52
C-3Cl,4Me	k_1	1.18	1.24 (*M*)	1.13 (*M*)	1.05 (*M*)	1.14 (*M*)	1.07 (*M*)	0.87 (*M*)	1.22 (*M*)
	k_2		1.35 (*P*)	1.25 (*P*)	1.26 (*P*)	1.24 (*P*)	1.18 (*P*)	1.15 (*P*)	1.81 (*P*)
	α		1.09	1.11	1.20	1.09	1.11	1.32	1.49
A-3,5diMe	k_1	1.30	1.26 (*M*)	1.30	1.08 (*M*)	1.11 (*M*)	1.15 (*P*)	0.88 (*M*)	0.99 (*M*)
	k_2		1.50 (*P*)	1.30	1.64 (*P*)	1.29 (*P*)	1.22 (*M*)	1.27 (*P*)	2.19 (*P*)
	α		1.19	1.00	1.52	1.16	1.06	1.44	2.21
iA-3,5diMe	k_1	1.07	1.08 (*M*)	1.08 (*M*)	1.18 (*M*)	1.05 (*M*)	1.04	0.99 (*M*)	1.11 (*M*)
	k_2		1.31 (*P*)	1.13 (*P*)	1.97 (*P*)	1.22 (*P*)	1.04	1.54 (*P*)	2.56 (*P*)
	α		1.21	1.05	1.67	1.16	1.00	1.56	2.31
A-5Cl,2Me	k_1	1.60	1.95 (*M*)	1.93 (*M*)	1.59 (*M*)	1.71 (*M*)	1.71 (*M*)	1.57 (*M*)	1.66
	k_2		2.14 (*P*)	2.36 (*P*)	2.50 (*P*)	1.88 (*P*)	2.10 (*P*)	2.33 (*P*)	1.66
	α		1.10	1.22	1.57	1.10	1.23	1.48	1.00
iA-3Cl,-Me	k_1	1.15	1.31	1.23	1.06 (*M*)	1.16	1.10	0.88 (*M*)	1.05 (*M*)
	k_2		1.31	1.23	1.23 (*P*)	1.16	1.10	1.00 (*P*)	1.30 (*P*)
	α		1.00	1.00	1.16	1.00	1.00	1.14	1.24

[1] Chiral column (selector): Lux Cellulose-1 (coated C-3,5diMe), Lux Cellulose-2 (coated C-3Cl,4Me), Lux Amylose-1 (coated A-3,5diMe); Lux i-Amylose-1 (immobilized A-3,5diMe) (iA-3,5diMe); Lux Amylose-2 (coated A-5Cl,2Me); Lux i-Amylose-3 (immobilized A-3Cl,5Me) (iA-3Cl,5Me).

Table S3. Retention and selectivity of 4,4′-bipyridines **1**-**8** on coated Lux Cellulose-1 (C-3,5diMe) by using Hex/IPA 90:10 (*mix A*), Hex/IPA/MeOH 90:5:5 (*mix B*) and MeOH (*mix C*) as mobile phases (*FR* 0.8 ml/min, T = 25°C) (the absolute configuration of eluted enantiomers is reported in brackets).

Chiral column (MP)	k_1, k_2, α	**1**	**2**	**3**	**4**	**5**	**6**	**7**	**8**
Lux Cellulose-1 (A)	k_1	1.28	1.60 (*P*)	1.15 (*M*)	1.28 (*M*)	1.44 (*P*)	1.09 (*M*)	1.14 (*M*)	1.27 (*M*)
	k_2		1.77 (*M*)	1.31 (*P*)	4.75 (*P*)	1.55(*M*)	1.26 (*P*)	6.03 (*P*)	4.46 (*P*)
	α		1.11	1.14	3.71	1.07	1.16	5.27	3.52
Lux Cellulose-1 (B)	k_1	1.30	1.55 (*M*)	1.17 (*M*)	1.40 (*M*)	1.43 (*M*)	1.11 (*M*)	1.23 (*M*)	1.31 (*M*)
	k_2		1.73 (*P*)	1.32 (*P*)	3.80 (*P*)	1.60 (*P*)	1.25 (*P*)	4.55 (*P*)	4.40 (*P*)
	α		1.12	1.12	2.71	1.12	1.13	3.69	3.03
Lux Cellulose-1 (C)	k_1	0.66	0.89 (*M*)	1.13	0.85 (*M*)	0.79 (*M*)	0.98	0.86 (*M*)	0.68 (*M*)
	k_2		0.99 (*P*)	1.13	1.53 (*P*)	0.87 (*P*)	0.98	1.78 (*P*)	1.38 (*P*)
	α		1.11	1.00	1.80	1.10	1.00	2.08	2.02

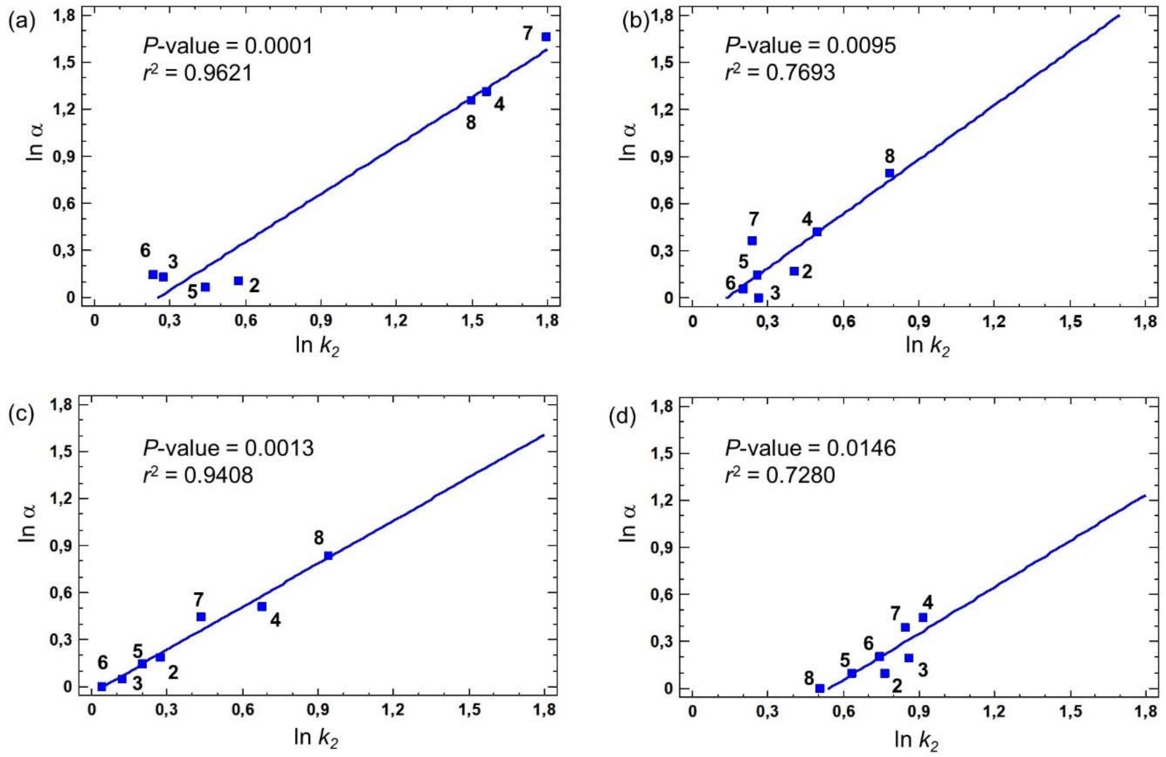

Figure S2. Linear regression analysis describing the relationships between ln α and ln k_2 for **2**-**8** on C-3,5diMe (a), A-3,5diMe (b), iA-3,5diMe (c), and A-5Cl,2Me (d) as chiral selectors.

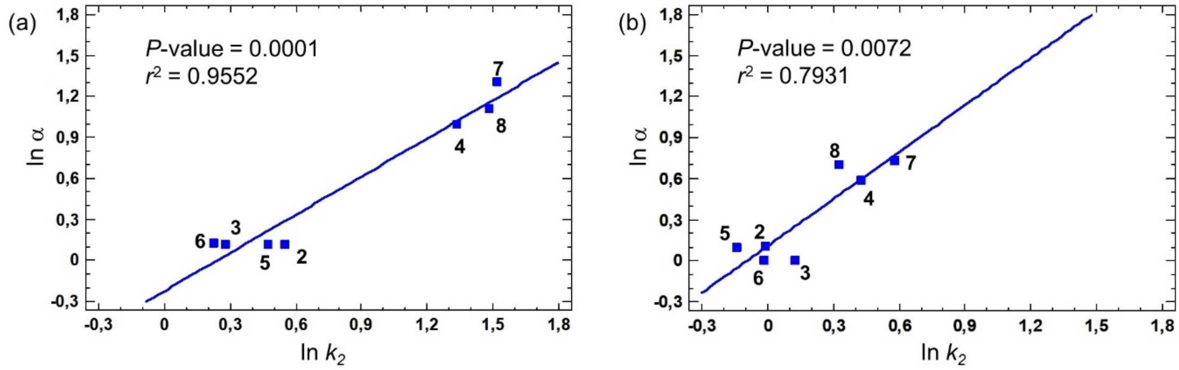

Figure S3. Linear regression analysis describing the relationships between ln α and ln k_2 for **2**-**8** on C-3,5diMe with *mix B* (a) and *mix C* (b) as MPs.

Table S4. Temperature dependence of retention factors and van't Hoff equations for 4,4′-bipyridines **2**-**8** on C-3,5diMe (Lux Cellulose-1), hex/IPA 90:10 (*mix A*), *FR* = 0.8 ml/min.

Bipy		Temperature (°C)							Regression equation	$r^{2\,a}$
		5	10	15	20	25	30	35		
**2**	k_1	2.41	2.14	1.91	1.77	1.60	1.46	1.33	$\ln k_1 = 1634.07x - 5.0089$	0.9988
	k_2	2.74	2.41	2.15	1.98	1.77	1.62	1.47	$\ln k_2 = 1723.94x - 5.2045$	0.9990
	α	1.14	1.13	1.12	1.12	1.11	1.11	1.10		
**3**	k_1	1.57	1.44	1.35	1.25	1.15	1.07	1.01	$\ln k_1 = 1255.03x - 4.0617$	0.9986
	k_2	1.87	1.69	1.57	1.44	1.31	1.21	1.12	$\ln k_2 = 1437.17x - 4.5427$	0.9985
	α	1.19	1.18	1.17	1.15	1.14	1.13	1.11		
**4**	k_1	2.01	1.79	1.61	1.46	1.28	1.17	1.07	$\ln k_1 = 1778.33x - 5.6989$	0.9988
	k_2	11.94	9.18	7.21	5.93	4.75	3.83	3.13	$\ln k_2 = 3725.19x - 10.9339$	0.9993
	α	5.94	5.14	4.48	4.08	3.71	3.26	2.91		
**5**	k_1	2.25	2.01	1.82	1.63	1.44	1.35	1.24	$\ln k_1 = 1697.92x - 5.2999$	0.9979
	k_2	2.45	2.18	1.98	1.76	1.55	1.45	1.32	$\ln k_2 = 1749.11x - 5.3972$	0.9974
	α	1.09	1.09	1.09	1.08	1.07	1.07	1.07		
**6**	k_1	1.48	1.35	1.27	1.16	1.09	1.01	0.94	$\ln k_1 = 1256.83x - 4.1312$	0.9993
	k_2	1.83	1.64	1.51	1.37	1.26	1.16	1.07	$\ln k_2 = 1494.23x - 4.7748$	0.9995
	α	1.23	1.21	1.20	1.18	1.16	1.15	1.13		
**7**	k_1	1.76	1.56	1.39	1.31	1.14	1.03	0.95	$\ln k_1 = 1725.33x - 5.6438$	0.9945
	k_2	16.28	12.53	9.51	7.49	6.03	4.76	3.79	$\ln k_2 = 4075.70x - 11.8767$	0.9993
	α	9.25	8.03	6.85	5.70	5.27	4.60	3.99		
**8**	k_1	1.92	1.74	1.59	1.43	1.27	1.15	1.04	$\ln k_1 = 1744.19x - 5.6070$	0.9961
	k_2	10.40	8.29	6.64	5.53	4.46	3.66	3.03	$\ln k_2 = 3453.73x - 10.0835$	0.9993
	α	5.41	4.76	4.19	3.87	3.52	3.19	2.91		

[a] r^2, correlation coefficient of van't Hoff plot ln *k* (1/T).

Table S5. Temperature dependence of retention factors and van't Hoff equations for 4,4′-bipyridines **2**-**8** on C-3Cl,4Me (Lux Cellulose-2) (hex/IPA 90:10 (*mix A*), *FR* = 0.8 ml/min.

Bipy		Temperature (°C)							Regression equation	$r^{2\,a}$
		5	10	15	20	25	30	35		
**2**	k_1	1.73	1.57	1.44	1.33	1.24	1.16	1.09	$\ln k_1 = 1294.12x - 4.1200$	0.9980
	k_2	1.92	1.73	1.58	1.46	1.35	1.26	1.18	$\ln k_2 = 1365.97x - 4.2738$	0.9981
	α	1.11	1.10	1.10	1.09	1.09	1.09	1.08		
**3**	k_1	1.56	1.42	1.31	1.22	1.13	1.07	1.02	$\ln k_1 = 1211.35x - 3.9267$	0.9942
	k_2	1.80	1.62	1.47	1.36	1.25	1.17	1.11	$\ln k_2 = 1363.78x - 4.3366$	0.9948
	α	1.15	1.14	1.13	1.12	1.11	1.10	1.09		
**4**	k_1	1.58	1.41	1.27	1.15	1.05	0.97	0.92	$\ln k_1 = 1544.96x - 5.1157$	0.9948
	k_2	2.02	1.77	1.56	1.40	1.26	1.15	1.07	$\ln k_2 = 1813.37x - 5.8352$	0.9961
	α	1.28	1.26	1.23	1.22	1.20	1.18	1.16		
**5**	k_1	1.59	1.45	1.33	1.23	1.14	1.08	1.02	$\ln k_1 = 1263.03x - 4.0902$	0.9965
	k_2	1.76	1.59	1.46	1.34	1.24	1.16	1.10	$\ln k_2 = 1339.30x - 4.2637$	0.9967
	α	1.11	1.10	1.09	1.09	1.09	1.08	1.08		
**6**	k_1	1.48	1.34	1.23	1.15	1.07	1.00	0.96	$\ln k_1 = 1208.09x - 3.9724$	0.9923
	k_2	1.72	1.54	1.40	1.29	1.18	1.10	1.05	$\ln k_2 = 1397.57x - 4.5021$	0.9934
	α	1.17	1.15	1.13	1.12	1.11	1.10	1.09		
**7**	k_1	1.31	1.17	1.04	0.96	0.87	0.81	0.77	$\ln k_1 = 1518.46x - 5.2101$	0.9918
	k_2	1.92	1.67	1.45	1.29	1.15	1.04	0.97	$\ln k_2 = 1953.40x - 6.3911$	0.9952
	α	1.46	1.43	1.39	1.35	1.32	1.29	1.25		
**8**	k_1	1.94	1.68	1.48	1.34	1.22	1.10	1.01	$\ln k_1 = 1814.62x - 5.8859$	0.9971
	k_2	3.20	2.71	2.33	2.04	1.81	1.60	1.44	$\ln k_2 = 2229.55x - 6.8760$	0.9980
	α	1.65	1.61	1.57	1.53	1.49	1.46	1.43		

[a] r^2, correlation coefficient of van't Hoff plot ln k (1/T).

Table S6. Temperature dependence of retention factors and van't Hoff equations for 4,4′-bipyridines **2**-**8** on C-3,5diMe (Lux Cellulose-1), hex/IPA/MeOH 90:5:5 (*mix B*), *FR* = 0.8 ml/min.

Bipy		Temperature (°C)							Regression equation	$r^{2\,a}$
		5	10	15	20	25	30	35		
**2**	k_1	2.24	2.01	1.86	1.70	1.55	1.44	1.34	$\ln k_1 = 1443.27x - 5.4392$	0.9995
	k_2	2.55	2.29	2.09	1.91	1.73	1.60	1.47	$\ln k_2 = 1535.18x - 4.5906$	0.9998
	α	1.14	1.14	1.12	1.12	1.12	1.11	1.10		
**3**	k_1	1.58	1.44	1.35	1.22	1.17	1.11	1.04	$\ln k_1 = 1159.21x - 3.7254$	0.9944
	k_2	1.80	1.64	1.52	1.38	1.32	1.24	1.15	$\ln k_2 = 1228.16x - 3.8418$	0.9960
	α	1.14	1.14	1.13	1.13	1.12	1.12	1.11		
**4**	k_1	2.17	1.91	1.73	1.53	1.40	1.28	1.18	$\ln k_1 = 1711.29x - 5.3939$	0.9979
	k_2	8.09	6.72	5.45	4.51	3.80	3.21	2.74	$\ln k_2 = 3065.97x - 8.9387$	0.9990
	α	3.73	3.52	3.15	2.95	2.71	2.51	2.32		
**5**	k_1	2.06	1.84	1.71	1.56	1.43	1.32	1.23	$\ln k_1 = 1441.04x - 4.4695$	0.9993
	k_2	2.33	2.09	1.92	1.75	1.60	1.47	1.36	$\ln k_2 = 1513.69x - 4.6023$	0.9998
	α	1.13	1.14	1.12	1.12	1.12	1.11	1.10		
**6**	k_1	1.56	1.41	1.31	1.19	1.11	1.05	0.99	$\ln k_1 = 1268.34x - 4.1323$	0.9940
	k_2	1.79	1.62	1.50	1.36	1.25	1.19	1.11	$\ln k_2 = 1349.61x - 4.2822$	0.9957
	α	1.15	1.15	1.14	1.14	1.13	1.13	1.12		
**7**	k_1	1.90	1.68	1.51	1.35	1.23	1.13	1.05	$\ln k_1 = 1687.41x - 5.4392$	0.9976
	k_2	10.51	8.55	6.80	5.53	4.55	3.76	3.14	$\ln k_2 = 3415.50x - 9.9314$	0.9992
	α	5.52	5.08	4.51	4.08	3.69	3.34	3.00		
**8**	k_1	2.02	1.78	1.61	1.45	1.31	1.21	1.10	$\ln k_1 = 1685.05x - 5.3688$	0.9995
	k_2	7.68	6.38	5.25	4.40	3.71	3.16	2.69	$\ln k_2 = 2951.11x - 8.5782$	0.9995
	α	3.80	3.58	3.26	3.03	2.82	2.62	2.44		

[a] r^2, correlation coefficient of van't Hoff plot ln *k* (1/T).

Table S7. Temperature dependence of retention factors and van't Hoff equations for 4,4′-bipyridines **2**-**8** on C-3,5diMe (Lux Cellulose-1), MeOH 100% (*mix C*), *FR* = 0.8 ml/min.

Bipy		Temperature (°C)							Regression equation	$r^{2\,a}$
		5	10	15	20	25	30	35		
**2**	k_1	1.16	1.08	1.01	0.95	0.89	0.84	0.79	$\ln k_1 = 1073.05x - 3.7106$	0.9993
	k_2	1.33	1.23	1.14	1.06	0.99	0.92	0.87	$\ln k_2 = 1202.33x - 4.0405$	0.9992
	α	1.15	1.13	1.12	1.11	1.11	1.10	1.09		
**3**	k_1	1.47	1.37	1.29	1.21	1.13	1.07	1.01	$\ln k_1 = 1046.63x - 3.3797$	0.9990
	k_2	1.47	1.37	1.29	1.21	1.13	1.07	1.01		
	α	1.00	1.00	1.00	1.00	1.00	1.00	1.00		
**4**	k_1	1.13	1.05	0.98	0.91	0.85	0.80	0.75	$\ln k_1 = 1145.22x - 3.9989$	0.9992
	k_2	2.45	2.17	1.92	1.71	1.53	1.36	1.23	$\ln k_2 = 1941.05x - 6.0847$	0.9993
	α	2.17	2.07	1.97	1.87	1.80	1.71	1.63		
**5**	k_1	1.03	0.96	0.90	0.84	0.79	0.74	0.71	$\ln k_1 = 1061.59x - 3.7911$	0.9989
	k_2	1.15	1.07	0.99	0.92	0.87	0.81	0.76	$\ln k_2 = 1155.04x - 4.0158$	0.9993
	α	1.12	1.11	1.10	1.10	1.10	1.09	1.08		
**6**	k_1	1.26	1.18	1.12	1.05	0.98	0.93	0.89	$\ln k_1 = 989.20x - 3.3251$	0.9982
	k_2	1.26	1.18	1.12	1.05	0.98	0.93	0.89		
	α	1.00	1.00	1.00	1.00	1.00	1.00	1.00		
**7**	k_1	1.16	1.07	1.00	0.92	0.86	0.80	0.75	$\ln k_1 = 1231.07x - 4.2784$	0.9992
	k_2	3.04	2.65	2.31	2.02	1.78	1.56	1.38	$\ln k_2 = 2216.74x - 6.8583$	0.9992
	α	2.62	2.47	2.31	2.19	2.08	2.00	1.84		
**8**	k_1	0.90	0.84	0.78	0.72	0.68	0.63	0.60	$\ln k_1 = 1163.35x - 4.2880$	0.9992
	k_2	2.22	1.96	1.73	1.53	1.38	1.22	1.09	$\ln k_2 = 1998.36x - 6.3882$	0.9990
	α	2.46	2.34	2.22	2.11	2.02	1.92	1.82		

[a] r^2, correlation coefficient of van't Hoff plot ln *k* (1/T).

Table S8. Thermodynamic quantities calculated from the van't Hoff plots for 4,4′-bipyridines **2**-**8** on C-3,5-diMe and C-3Cl,4Me under normal phase (NP) and polar organic (PO) elution modes (*FR* = 0.8 ml/min).

Bipy[1]		CSP	MP[2]	ΔH [kJ/mol]	ΔS* [J/(Kmol)]	$\Delta G_{298\ K}$ [kJ/mol]	ΔΔH [kJ/mol]	ΔΔS [J/(Kmol)]	$\Delta\Delta G_{298\ K}$ [kJ/mol]
**2**	*P*	C-3,5diMe	A	-13.59	-41.65	-1.17	-0.74	-1.62	-0.26
	M			-14.33	-43.27	-1.43			
	M	C-3Cl,4Me	A	-10.76	-34.25	-0.55	-0.60	-1.28	-0.22
	P			-11.36	-35.53	-0.77			
	M	C-3,5diMe	B	-12.00	-36.51	-1.11	-0.76	-1.66	-0.27
	P			-12.76	-38.17	-1.38			
	M	C-3,5diMe	C	-8.92	-30.85	0.28	-1.08	-2.74	-0.26
	P			-10.00	-33.59	0.02			
**3**	*M*	C-3,5diMe	A	-10.43	-33.77	-0.36	-1.52	-4.00	-0.33
	P			-11.95	-37.77	-0.69			
	M	C-3Cl,4Me	A	-10.07	-32.65	-0.34	-1.27	-3.41	-0.25
	P			-11.34	-36.06	-0.59			
	M	C-3,5diMe	B	-9.64	-30.97	-0.41	-0.57	-0.97	-0.28
	P			-10.21	-31.94	-0.69			
	M,P	C-3,5diMe	C	-8.70	-28.10	-0.32	0	0	0
**4**	*M*	C-3,5diMe	A	-14.78	-47.38	-0.65	-16.19	-43.53	-3.21
	P			-30.97	-90.91	-3.86			
	M	C-3Cl,4Me	A	-12.84	-42.53	-0.16	-2.24	-5.99	-0.45
	P			-15.08	-48.52	-0.61			
	M	C-3,5diMe	B	-14.23	-44.85	-0.86	-11.26	-29.47	-2.47
	P			-25.49	-74.32	-3.33			
	M	C-3,5diMe	C	-9.52	-33.25	0.39	-6.62	-17.34	-1.45
	P			-16.14	-50.59	-1.06			
**5**	*P*	C-3,5diMe	A	-14.11	-44.06	-0.97	-0.43	-0.81	-0.19
	M			-14.54	-44.87	-1.16			
	M	C-3Cl,4Me	A	-10.50	-34.01	-0.36	-0.63	-1.44	-0.20
	P			-11.13	-35.45	-0.56			
	M	C-3,5diMe	B	-11.98	-37.16	-0.90	-0.60	-1.10	-0.27
	P			-12.58	-38.26	-1.17			
	M	C-3,5diMe	C	-8.83	-31.52	0.57	-0.77	-1.87	-0.21
	P			-9.60	-33.39	0.36			
**6**	*M*	C-3,5diMe	A	-10.45	-34.35	-0.21	-1.97	-5.35	-0.37
	P			-12.42	-39.70	-0.58			
	M	C-3Cl,4Me	A	-10.04	-33.03	-0.19	-1.58	-4.40	-0.27
	P			-11.62	-37.43	-0.46			
	M	C-3,5diMe	B	-10.54	-34.36	-0.29	-0.68	-1.24	-0.31
	P			-11.22	-35.60	-0.60			
	M,P	C-3,5diMe	C	-8.22	-27.65	0.02	0	0	0
**7**	*M*	C-3,5diMe	A	-14.34	-46.92	-0.35	-19.55	-51.83	-4.10
	P			-33.89	-98.75	-4.45			
	M	C-3Cl,4Me	A	-12.63	-43.32	0.29	-3.62	-9.82	-0.69
	P			-16.24	-53.14	-0.40			
	M	C-3,5diMe	B	-14.03	-45.22	-0.55	-14.37	-37.35	-3.23
	P			-28.40	-82.57	-3.78			
	M	C-3,5diMe	C	-10.24	-35.57	0.36	-8.19	-21.45	-1.79
	P			-18.43	-57.02	-1.43			
**8**	*M*	C-3,5diMe	A	-14.50	-46.62	-0.60	-14.22	-37.22	-3.12
	P			-28.72	-83.84	-3.72			
	M	C-3Cl,4Me	A	-15.09	-48.94	-0.50	-3.45	-8.23	-1.00
	P			-18.54	-57.17	-1.50			
	M	C-3,5diMe	B	-14.01	-44.64	-0.70	-10.53	-26.68	-2.57
	P			-24.54	-71.32	-3.27			
	M	C-3,5diMe	C	-9.67	-35.65	0.96	-6.94	-17.46	-1.73
	P			-16.61	-53.11	-0.77			

[1] Absolute configuration of the eluted peaks is reported. [2] Hex/IPA 90:10 (A); Hex/IPA/MeOH 90:5:5 (B); MeOH 100% (C).

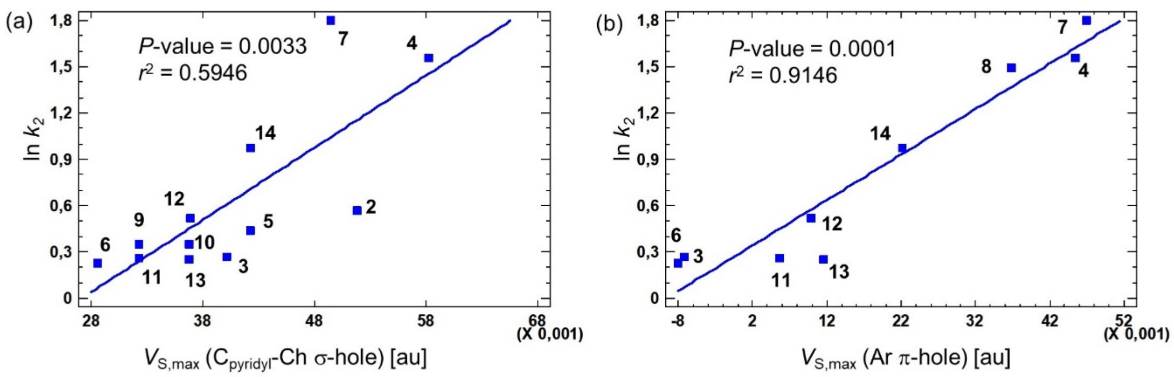

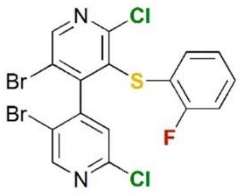
9

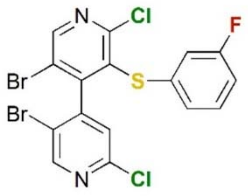
10

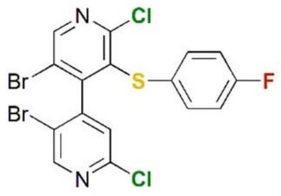
11

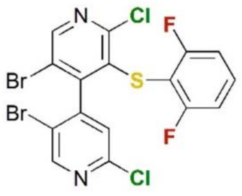
12

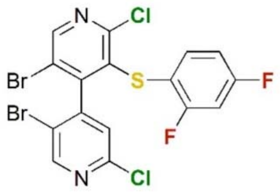
13

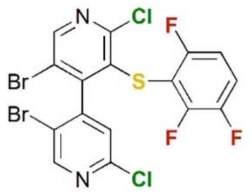
14

Figure S4. Linear regression analysis describing the relationships between ln k_2 (C-3,5diMe, *mix A*) and $V_{S,max}$ ($C_{pyridyl}$-Ch σ-hole) [au] (compounds **2-7** and **9-14**) (a) and $V_{S,max}$ (Ar π-hole) [au] (compounds **3**, **4**, **6-8**, and **11-14**) (b).
